# Tailored resection for persistent extramural vascular invasion in locally advanced rectal cancers

**DOI:** 10.1111/codi.17234

**Published:** 2024-11-12

**Authors:** Devesh S. Ballal, Ankit Sharma, Yogesh Bansod, Suman K. Ankathi, Mufaddal Kazi, Ashwin Desouza, Avanish P. Saklani

**Affiliations:** ^1^ Division of Colon and Rectal Surgery Advocate Lutheran General Hospital Park Ridge Illinois USA; ^2^ Division of Colo‐Rectal and Peritoneal Surface Oncology, Department of Surgical Oncology Tata Memorial Hospital, Homi Bhabha National Institute Mumbai India; ^3^ Department of Radiodiagnosis Tata Memorial Hospital, Homi Bhabha National Institute Mumbai India

**Keywords:** beyond TME, EMVI, local recurrence, rectal cancer

## Abstract

**Background:**

Extramural vascular invasion (EMVI) is a bad prognostic feature in rectal cancer and cancers that remain EMVI positive after neoadjuvant therapy are at high risk for having involved circumferential resection margins. Conventional total mesorectal excision (TME) resections are inadequate in such cases and often lead to positive margins.

**Methods:**

We propose a technique for the surgical management of locally advanced tumours with persistent EMVI after neoadjuvant therapy. Ten such tumours were resected using a “beyond TME” (b‐TME) approach with or without lateral pelvic lymph node dissection or seminal vesical excision.

**Results:**

A b‐TME approach, customized to the anatomy of the tumour allowed for an R0 resection with a negative circumferential resection margin (CRM) in all 10 cases.

**Conclusion:**

A tailored b‐TME approach can achieve good results in cases at high risk for CRM involvement.

## INTRODUCTION

Extramural vascular invasion (EMVI) is an adverse prognostic sign in rectal cancer and is known to be associated with higher rates of distant metastasis [1]. However, a worrisome fact that is often overlooked is that EMVI not only portends an increased susceptibility to distant spread but is also associated with much higher rates of local recurrence and margin positivity after resection [2]. Growth of cancer into blood vessels increases the likelihood of tumour deposits that threaten the circumferential resection margin (CRM) as well the likelihood of pathologically involved lateral pelvic nodes [3], both of which require more aggressive local excisions. It is thus important to standardize and adopt more aggressive surgery beyond the conventionally performed total mesorectal excision (TME) for patients with persistent EMVI after neoadjuvant therapy who have a high risk of local failure.

## METHODS

We propose a technique for the surgical management of locally advanced tumours with persistent EMVI after neoadjuvant therapy. Ten such cases with persistent EMVI despite neoadjuvant therapy were successfully managed with a “beyond‐TME” (b‐TME) approach with or without lateral pelvic lymph node dissection or seminal vesicle excision, in which the dissection plane is carried beyond the conventionally followed plane to allow for optimal margins at sites of threatened CRM depending on the anatomy of the EMVI in the patient (Video 1).

**VIDEO 1:** Tailored surgical approach for rectal cancer with persistent EMVI. Video content can be viewed at https://onlinelibrary.wiley.com/doi/10.1111/codi.17234

The resection is tailored based on both baseline and posttreatment magnetic resonance imaging (MRI) scans, taking into consideration the location of the EMVI (lateral/anterior or posterior), presence of tumour deposits threatening the CRM and lateral pelvic nodes. The initial procedure of pedicle dissection and posterior dissection of TME plane is the same as what is done during standard TME resections and has been described in previously published videos [4, 5]. The technique for extended resections has been published previously by the authors [6] and is described in brief below:

If an extended dissection is required laterally, an initial peritoneal incision is made laterally over the iliac vessels and then extended anteriorly till the vas deferens/round ligament is encountered to mobilize the ureter along with the uretero‐hypogastric fascia. Dissection along the obliterated umbilical vessels is then done to develop the medial and lateral paravesical spaces. Depending on the location of EMVI on the preoperative MRI, the hypogastric nerve of one side is sacrificed and dissection proceeds posterolaterally to identify the S3/4 nerve roots and then proceeds to the pelvic floor. On the side of extended resection, the middle rectal artery is clipped at its origin from the internal iliac, sacrificing the autonomic nerves that would usually be preserved in standard TME resections. In cases where the EMVI is confluent with the lateral pelvic nodes, the inferior vesical vessels or even the internal iliac vessels on that side may be ligated to achieve an en‐bloc removal without entering the tumour.

Anterior dissection is tailored as per quadrant of involvement and involves resection of one plane beyond the tumour. Usually, the Denonviliers' fascia is excised with the rectum in cases with close or threatened anterior CRM, but in cases with frank invasion of the Denoviliers' on MRI, the seminal vesicles are encompassed in the resection to achieve negative margins. Circumferential mobilization with extended resection at sites of threatened margin facilitates rectal transection at a level appropriate for the particular tumour.

## RESULTS

All 10 patients underwent uncomplicated b‐TME surgeries without the development of any major (Clavien‐Dindo Grade > 3) complications. A complete resection with negative margins (R0) resection was obtained in all patients. The tumour and patient details are described in Table 1.

**TABLE 1:** Tumour characteristics and operative details.

S. no	Age	Sex	Location in rectum	Neoadjuvant therapy	Location of EMVI	Surgery done	Margins	Nodes
1	66	F	Lower third	4# PRRT	Right lateral	Lap LAR + right PLND	Free	2/26
2	55	M	Mid‐third	LCRT	Left anterolateral	Lap LAR + left PLND	Free	0/19
3	67	M	Lower third	SCRT + 4# FOLFOX	Left anterolateral	Lap APR + B/L SV excision	Free	1/22
4	78	M	Mid‐third	SCRT	Right lateral	Lap APR + right PLND + B/L SV excision	Free	7/17
5	58	M	Lower‐mid	LCRT	Left lateral	Robotic LAR + left hypogastric nerve excision	Free	4/14
6	33	M	Lower third	LCRT	Right lateral + posterior	Lap APR + right PLND + prostate shave + presacral fascia excision + S5 sacrectomy	Free	11/26
7	39	F	Lower third	SCRT + 5# FOLFIRINOX	Right lateral	Lap LAR with B/L PLND	Free	10/36
8	58	M	Lower third	LCRT	Left lateral	LAR with left hypogastric nerve excision	Free	4/14
9	51	M	Mid‐third	SCRT + 5# FOLFIRINOX	Right lateral	LAR with right hypogastric nerve excision	Free	0/7
10	34	M	Lower third	SCRT + 4# CAPOX	Right posterolateral	LAR with right S2–S4 nerve resection	Free	1/16

Abbreviations: APR, abdominoperineal resection; B/L, bilateral; EMVI, extramural vascular invasion; FOLFIRINOX/CAPOX/FOLFOX, chemotherapy regimens; Lap, laparoscopic; LAR, low anterior resection; LCRT, long‐course chemoradiotherapy; PLND, pelvic lymph node dissection; PRRT, peptide receptor radionucleotide therapy; SCRT, short‐course radiotherapy; SV, seminal vesicle.

## DISCUSSION

MRI‐detected EMVI is a well‐documented adverse prognostic sign in rectal cancer and is not only associated with a higher incidence of distant metastasis but also leads to an increase in the rate of local recurrences and margin‐positive resections [1, 2, 7]. EMVI is also associated with the presence of tumour deposits, EMVI that persists after neoadjuvant therapy is associated with poorer disease‐free survival and increases the risk of incomplete resections with a study showing an almost twofold increase in the rate of R1 resection (11.7% vs. 6.9%, *p* = 0.006) [2]. The surgical management of locally advanced rectal cancers with CRM involvement is not well defined. This subset of cancers has a very high risk of local recurrence, with studies showing that anteriorly‐based low rectal cancers with EMVI detected on MRI have rates of pCRM involvement of up to 60% [8]. The planning of surgical resections for these cancers is challenging and there the question of whether to use the pretreatment MRI or post‐neoadjuvant therapy MRI for surgical planning remains unanswered.

The dual blood supply of the rectum with a high degree of variability in the anatomy of the middle rectal artery makes the anatomical challenges presented unique: tumours that grow along the superior rectal vessels are the easiest to manage and a conventional TME often suffices; however, tumours growing along the middle and inferior rectal vessels pose unique challenges. The anatomy of the middle rectal vessels is extremely variable, maybe absent in up to 10%–30% of cadavers [9, 10] and is thought to be the route of cancerous involvement of the lateral pelvic nodes [11]. Growth of cancer into these vessels leads to a threatened CRM laterally, as these vessels traverse through the pelvic plexi at the level of S2–S4 as they communicate with the internal iliac vessels. The anatomic variations of the rectal vessels lead to multiple possible locations of threatened CRM, most often either anterior along the seminal vesicle/Denonviliers' fascia or laterally. A good understanding of pelvic anatomy allows for a tailored b‐TME excision to be performed depending on the tumour anatomy of the patient and should allow for lower rates of pathological CRM (pCRM) positivity. While our results are encouraging, with an R0 resection achieved in all patients, it is important to understand that dissection outside the TME plane leads to disruption of the autonomic supply to the pelvic viscera and patients need to be counselled regarding potential erectile dysfunction, retrograde ejaculation, and urinary retention preoperatively.

It is interesting to note that while much emphasis has been placed on total neoadjuvant therapy (TNT) to reduce rates of distant metastasis, efforts to reduce rates of local recurrence in nonresponders to this approach (about 20% of patients [12]) have not developed in parallel. TNT also brings with it numerous questions regarding the type of chemotherapy regimen to use and type of radiation (short‐ or long‐course) to be employed. While the response of EMVI to neoadjuvant therapy is prognostic (with patients in whom EMVI disappears having better outcomes [13]) it is important to understand that the EMVI detected on MRI is one of the main risk factors for local recurrence.

It is important to understand that systemic therapy cannot salvage inadequate surgical resection and knowledge of beyond‐TME resections to manage cancers with persistent EMVI and threatened margins post‐neoadjuvant therapy is sorely needed.

While the short‐term oncological outcomes of this technique appear promising, a longer follow‐up is required to know the true impact of these extended resections in this high‐risk cohort of rectal cancer patients. The disruption of the autonomic supply to pelvic organs by extended resections does lead to higher rates of urinary retention and sexual dysfunction, which is usually self‐limiting, provided the autonomic supply of one side is preserved and seminal vesicles are not removed.

In conclusion, more extensive surgery is needed to reduce the high local failure rates in locally advanced rectal cancer cases with EMVI. Using preoperative MRI to plan a standardized beyond‐TME resection allows for adequate margins and can lower the rates of pCRM positivity in these high‐risk cases.

## AUTHOR CONTRIBUTIONS

**Devesh S. Ballal:** Investigation; writing – original draft; methodology; writing – review and editing; software; data curation; formal analysis. **Ankit Sharma:** Methodology; investigation; writing – review and editing; software; data curation; formal analysis; validation. **Yogesh Bansod:** Investigation; software; data curation; writing – review and editing. **Suman K. Ankathi:** Investigation; methodology; formal analysis; supervision; writing – review and editing. **Mufaddal Kazi:** Investigation; writing – review and editing; project administration; supervision. **Ashwin Desouza:** Writing – review and editing; investigation; project administration; supervision. **Avanish P. Saklani:** Conceptualization; investigation; writing – review and editing; supervision; project administration; validation; visualization.

## CONFLICT OF INTEREST STATEMENT

The authors have no conflicts of interest to disclose or sources of financial support to declare.

## ETHICS STATEMENT

This study was carried out in concordance with the ethical standards set by the institutional ethics committee and being a retrospective review of previously collected data, without any patient identifiers being disclosed, a waiver for the need of patient consent was granted.

## Data Availability

The data that support the findings of this study are available from the corresponding author upon reasonable request.
